# Impact of Inversion Time for FLAIR Acquisition on the T2-FLAIR Mismatch Detectability for *IDH*-Mutant, Non-CODEL Astrocytomas

**DOI:** 10.3389/fonc.2020.596448

**Published:** 2021-01-14

**Authors:** Manabu Kinoshita, Hideyuki Arita, Masamichi Takahashi, Takehiro Uda, Junya Fukai, Kenichi Ishibashi, Noriyuki Kijima, Ryuichi Hirayama, Mio Sakai, Atsuko Arisawa, Hiroto Takahashi, Katsuyuki Nakanishi, Naoki Kagawa, Kouichi Ichimura, Yonehiro Kanemura, Yoshitaka Narita, Haruhiko Kishima

**Affiliations:** ^1^ Department of Neurosurgery, Osaka University Graduate School of Medicine, Suita, Japan; ^2^ Department of Neurosurgery, Takatsuki General Hospital, Takatsuki, Japan; ^3^ Department of Neurosurgery and Neuro-Oncology, National Cancer Center Hospital, Tokyo, Japan; ^4^ Department of Neurosurgery, Osaka City University Graduate School of Medicine, Osaka, Japan; ^5^ Department of Neurological Surgery, Wakayama Medical University, Wakayama, Japan; ^6^ Department of Neurosurgery, Osaka City General Hospital, Osaka, Japan; ^7^ Department of Diagnostic Radiology, Osaka International Cancer Institute, Osaka, Japan; ^8^ Department of Radiology, Osaka University Graduate School of Medicine, Suita, Japan; ^9^ Division of Brain Tumor Translational Research, National Cancer Center Research Institute, Tokyo, Japan; ^10^ Department of Biomedical Research and Innovation, Institute for Clinical Research, National Hospital Organization Osaka National Hospital, Osaka, Japan

**Keywords:** glioma, T2-weighted image, fluid-attenuated inversion recovery, *IDH*-mutation, radiogenomics

## Abstract

The current research tested the hypothesis that inversion time (TI) shorter than 2,400 ms under 3T for FLAIR can improve the diagnostic accuracy of the T2-FLAIR mismatch sign for identifying *IDH*mt, non-CODEL astrocytomas. We prepared three different cohorts; 94 MRI from 76 *IDH*mt, non-CODEL Lower-grade gliomas (LrGGs), 33 MRI from 31 LrGG under the restriction of FLAIR being acquired with TI < 2,400 ms for 3T or 2,016 ms for 1.5T, and 112 MRI from 112 patients from the TCIA/TCGA dataset for LrGG. The presence or absence of the “T2-FLAIR mismatch sign” was evaluated, and we compared diagnostic accuracies according to TI used for FLAIR acquisition. The T2-FLAIR mismatch sign was more frequently positive when TI was shorter than 2,400 ms under 3T for FLAIR acquisition (*p* = 0.0009, Fisher’s exact test). The T2-FLAIR mismatch sign was positive only for *IDH*mt, non-CODEL astrocytomas even if we confined the cohort with FLAIR acquired with shorter TI (*p* = 0.0001, Fisher’s exact test). TCIA/TCGA dataset validated that the sensitivity, specificity, PPV, and NPV of the T2-FLAIR mismatch sign to identify *IDH*mt, non-CODEL astrocytomas improved from 31, 90, 79, and 51% to 67, 94, 92, and 74%, respectively and the area under the curve of ROC improved from 0.63 to 0.87 when FLAIR was acquired with shorter TI. We revealed that TI for FLAIR impacts the T2-FLAIR mismatch sign’s diagnostic accuracy and that FLAIR scanned with TI < 2,400 ms in 3T is necessary for LrGG imaging.

## Introduction

Molecular characterization of the tumor has become one of the necessary and essential procedures for taking care of patients suffering from WHO grades 2 and 3 gliomas, namely lower-grade gliomas (LrGG) (1). Among several genetic alterations found in gliomas, *IDH* mutation and *1p/19q* co-deletion status are the most clinically significant information, as these genetic alterations are critical prognostic markers (2–5). Various studies have attempted to non-invasively identify molecular characteristics of LrGG *via* radiological images in the hope to aid treatment decisions (6–12). Among these efforts, the T2-fluid-attenuated inversion recovery (FLAIR) mismatch sign has become one of the most prominent imaging features for *IDH*-mutant, *1p19q*-non-codeleted (*IDH*mt, non-CODEL) astrocytomas (13–15). The T2-FLAIR mismatch sign is defined as a “complete/near-complete hyperintense signal on T2-weighted image (T2WI) and relatively hypointense signal on FLAIR except for hyperintense peripheral rim” (13). Investigation of the Cancer Imaging Archive (TCIA)/Cancer Genome Atlas (TCGA) dataset discovered that the presence of the T2-FLAIR mismatch sign for LrGG was highly predictive of the tumor being *IDH*mt, non-CODEL. The low sensitivity of the T2-FLAIR mismatch sign, however, has been problematic (15).

The authors have recently reported that long T1 and T2 effects of *IDH*mt, non-CODEL astrocytomas were the possible cause of the T2-FLAIR mismatch sign and that inversion time (TI) for acquiring FLAIR could have an impact on the appearance of the T2-FLAIR mismatch (16). That research revealed that optimum TI seems to lie between 2,100 and 2,700 ms under 3T. The current study aimed to test the hypothesis that TI shorter than 2,400 ms under 3T for FLAIR, which is the average of 2,100 and 2,700 ms, can improve sensitivity without sacrificing specificity to detect the T2-FLAIR mismatch sign for identifying *IDH*mt, non-CODEL astrocytomas. The current research tested this hypothesis using a local dataset for LrGG and further validated on the TCGA/TCIA dataset for LrGG.

## Materials and Methods

### Patient Cohort

We carried out this research per the principles of the Helsinki Declaration. Internal ethical review boards of Osaka University Graduate School of Medicine (approval number: 13244) and all collaborative institutes approved this research. All patients provided written informed consent regarding this research.

**Figure 1:**
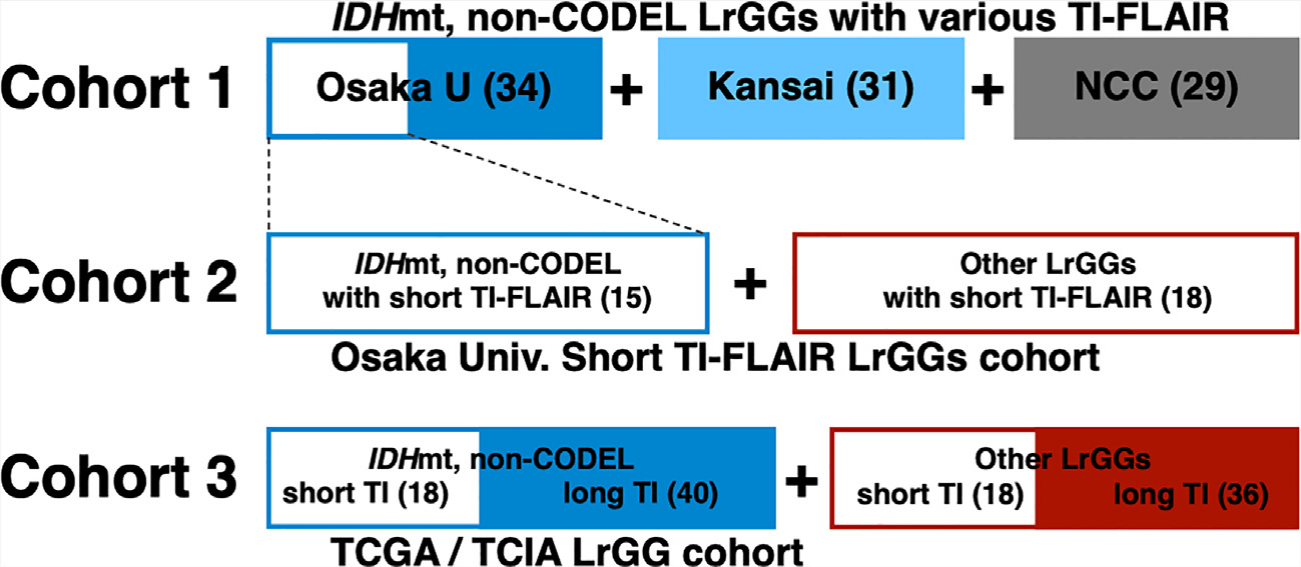
The entire cohort of the study is visualized. Cohort 1comprised of 94 MRI studies of histologically and molecularly confirmed *IDH*mt, non-CODEL LrGGs from the Kansai Molecular Diagnosis Network for CNS Tumors, which includes Osaka University Hospital, and the National Cancer Center Hospital. Cohort 2 comprised of 33 MRI studies of molecularly characterized LrGG from Osaka University Hospital under the restriction of FLAIR being acquired with TI < 2,400 ms (short TI-FLAIR) measured under 3T, which is equivalent to shorter than 2,016 ms under 1.5T. Cohort 3 comprised of 112 MRI studies from the TCIA/TCGA dataset for LrGG.

We prepared three different cohorts for this analysis (**Figure 1**). The first cohort (Cohort 1) comprised of 94 MRI studies from 76 histologically and molecularly confirmed *IDH*mt, non-CODEL LrGGs from six institutions. This cohort was built utilizing the Kansai Molecular Diagnosis Network for CNS Tumors, a region-based brain tumor tissue collection network that includes Osaka University Hospital (8, 9, 17), and an LrGG cohort provided from the National Cancer Center Hospital. **Supplementary Table 1** provides detailed information on this cohort. The second cohort (Cohort 2) comprised of 33 MRI studies from 31 histologically confirmed and molecularly characterized LrGG from a single institution (Osaka University Hospital). We collected the second cohort under the restriction of FLAIR being acquired with TI < 2,400 ms measured under 3T, which is equivalent to shorter than 2,016 ms under 1.5T. We state further rationale of this conversion in the following section, “Conversion of inversion time in 1.5T to 3T.” **Supplementary Table 2** provides detailed information about the second cohort. The third cohort (Cohort 3) comprised of 112 MRI studies from 112 patients from the TCIA/TCGA dataset for LrGG (15, 18, 19). **Supplementary Table 3** provides detailed information about the third cohort.

### Genetic Analysis

Two laboratories performed genetic analyses of the tissues: the Osaka National Hospital (ONH), Osaka, Japan, and the National Cancer Center Research Institute (NCC), Tokyo, Japan. Sanger sequencing or pyrosequencing assessed hotspot mutations of *IDH*1/2 (codon 132 of *IDH*1 and codon 172 of *IDH*2) and the *TERT* promoter (C228 and C250) at either lab. *1p/19q* co-deletion was detected either by copy number multiplex ligation-dependent probe amplification in a unified workflow at either lab or by fluorescence *in situ* hybridization (FISH) (20). We assigned the tumor as “non-CODEL” if the tumor was *TERT* promoter mutation-positive in cases where *1p/19q* was considered codeleted (as in case OUN-42 in **Supplementary Table 1**). The methylation status of the *MGMT* promoter was analyzed and assessed by qPCR at ONH or by pyrosequencing after bisulfite modification at NCC (20, 21). We obtained *IDH* and *TERT* promoter mutation and *1p19q* co-deletion statuses for the TCIA/TCGA dataset from the report by Ceccarelli *et al.* (22).

### Detection of T2-FLAIR Mismatch Sign

The presence or absence of the “T2-FLAIR mismatch sign” was evaluated in all cases by two independent readers (M.K. who has 20 and H.A. who has 15 years of experience in surgical neuro-oncology). A consensus meeting between the two readers finally determined the presence or absence of the “T2-FLAIR mismatch sign” strictly complying with the following criteria; complete/near-complete hyperintense signal on T2-weighted image (T2WI) and relatively hypointense signal on FLAIR except for hyperintense peripheral rim.

### Conversion of Inversion Time in 1.5T to 3T

We converted TI for FLAIR acquired under 1.5T to 3T to enable an even comparison of FLAIR acquired both in 1.5T and 3T MR scanners (Prisma or Aera; Siemens Healthcare, Erlangen, Germany). This process was necessary as TI is an MR acquisition parameter derived from T1 relaxation times, which is influenced by magnetic field strength. We converted T1-relaxation times measured on 1.5T MRI into estimated relaxation times measured at 3T by a conversion coefficient using data from a healthy volunteer. T1 relaxation times measured at 3T was plotted as a function of those measured at 1.5T. T1-relaxometry was achieved by first acquiring MP2RAGE images, then converting those images into T1-relaxation time maps with relaxometries performed *via* Bayesian inference modeling (Olea Nova+; Canon Medical Systems, Tochigi, Japan). MP2RAGE was acquired using repetition time (TR) = 5,000 ms; echo time (TE) = 3.86 ms; and inversion time (TI) = 935/2,820 ms. Measured values are compared in a voxel-wise manner, and the following conversion was obtained:

(1) $${\text{T}}_{1@3\text{T}}=1.19\times {\text{T}}_{1@1.5\text{T}}$$

As a result, the TI of 2,400 ms under 3T was equivalent to 2,016 ms under 1.5T.

### Statistical Analysis

We performed statistical analysis using Prism 8 for macOS (GraphPad Software, San Diego, CA, USA). Fisher’s exact test evaluated the statistical significance of contingency tables.

## Results

### TI of Shorter Than 2,400 ms for FLAIR Improves the Detectability of T2-FLAIR Mismatch Sign in *IDH*mt, Non-CODEL Astrocytomas (Study From Cohort 1)

**Figure 2:**
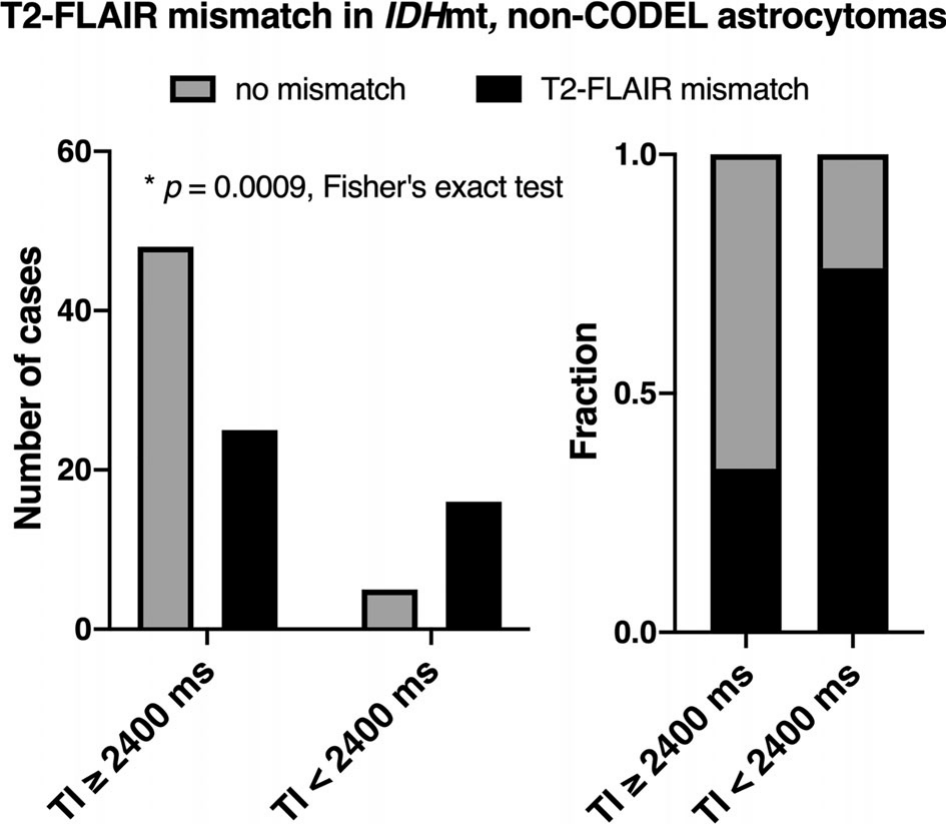
The figures show the frequencies of presence or absence of the T2-FLAIR mismatch sign as a function of TI for FLAIR acquisition confined within *IDH*mt, non-CODEL astrocytomas. The left panel shows the number of cases, and the right panel shows the frequency. The T2-FLAIR mismatch sign was more frequently positive when TI was shorter than 2,400 ms under 3T measurement for FLAIR acquisition (*p* = 0.0009, Fisher’s exact test).

**Figure 3:**
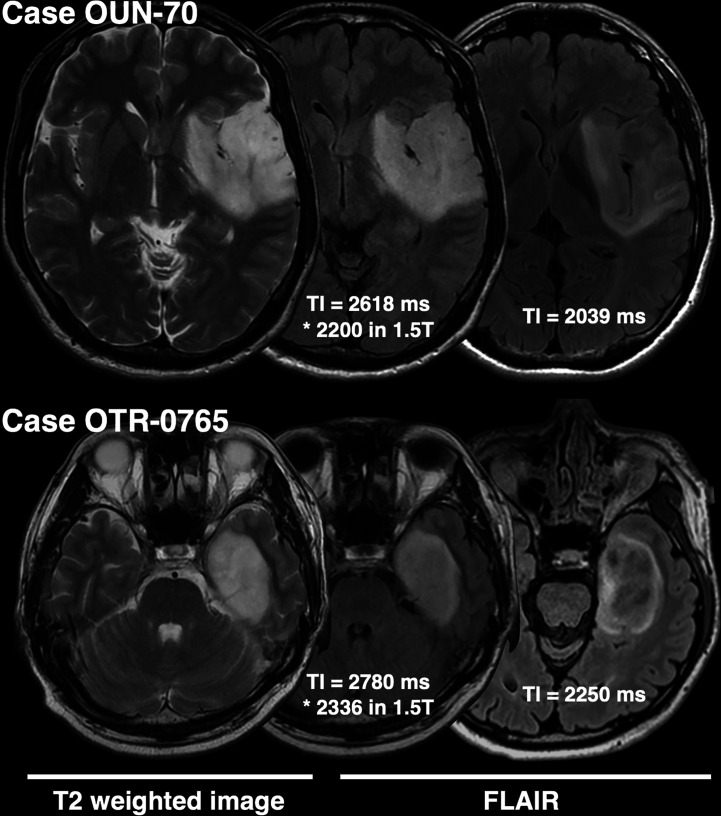
This figure shows two representative *IDH*mt, non-CODEL astrocytomas that had two FLAIR images scanned with different TIs. Both cases highlight the importance of TI for FLAIR acquisition in terms of visualization of the T2-FLAIR mismatch sign.

The frequency of presence or absence of the T2-FLAIR mismatch sign confined within *IDH*mt, non-CODEL astrocytomas, was assessed using the first cohort (**Supplementary Table 1**). The inter-rater reliability of the two readers evaluated by Cohen’s kappa coefficient was 0.60. Rater 2 showed a tendency to interpret the “T2-FLAIR mismatch sign” more strictly, which was true through cohorts 1 to 3. A consensus meeting further corrected the inter-rater disagreement. When FLAIR was acquired with TI shorter than 2,400 ms in 3T, the fraction of the T2-FLAIR mismatch sign positive astrocytomas increased to 0.76 compared to 0.34 in cases where TI longer than 2,400 ms was used (**Figure 2**, *p* = 0.0009, Fisher’s exact test). This result confirmed that lowering TI under 2,400 ms in 3T is favorable to increase the detectability of the T2-FLAIR mismatch sign. Some patients received more than two scans with FLAIR acquired using different TI. As shown in **Figure 3**, the ease of appreciating the T2-FLAIR mismatch sign was different between FLAIR with TI of shorter and longer than 2,400 ms.

### Presence of the T2-FLAIR Mismatch Sign With FLAIR of TI < 2,400 ms Is Specific for *IDH*mt, Non-CODEL Astrocytomas (Study From Cohort 2)

**Figure 4:**
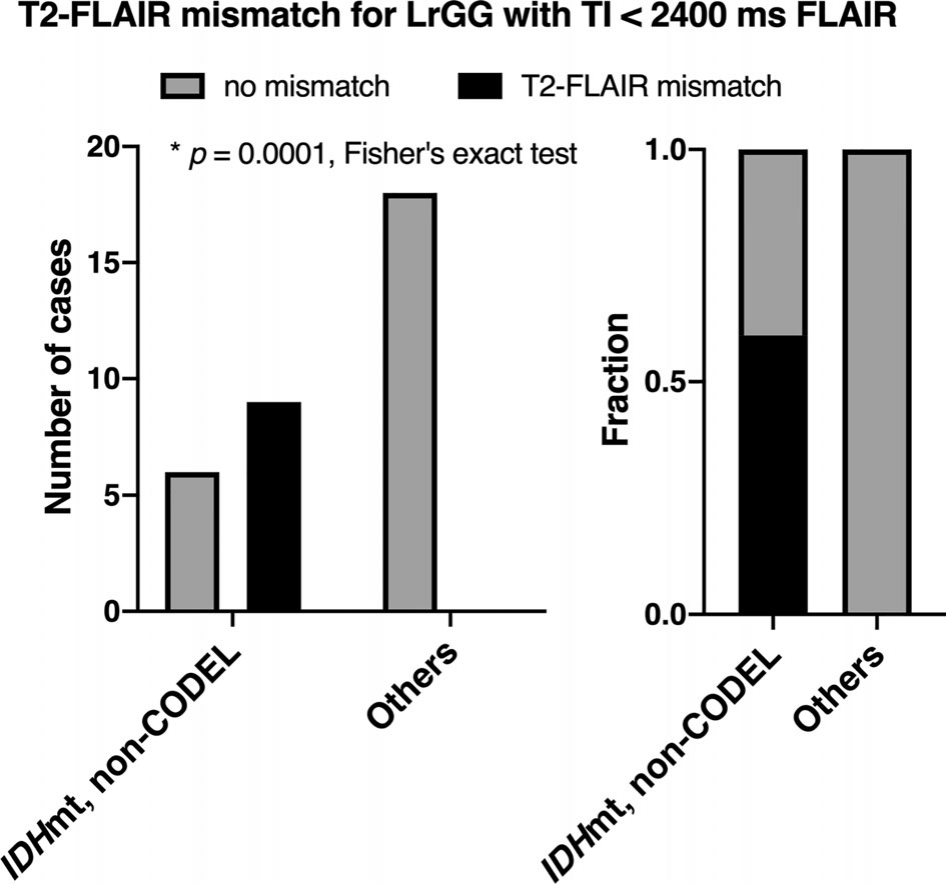
The figures show the frequencies of presence or absence of the T2-FLAIR mismatch sign among LrGGs with FLAIR acquired using TI shorter than 2,400 ms. The left panel shows the number of cases, and the right panel shows the frequency. The T2-FLAIR mismatch sign was positive only for *IDH*mt, non-CODEL astrocytomas (*p* = 0.0001, Fisher’s exact test).

The inter-rater reliability of the two readers assessed by Cohen’s kappa coefficient was 0.51. The specificity of the T2-FLAIR mismatch sign with FLAIR of TI < 2,400 ms was 100% along with a 60% sensitivity for identifying *IDH*mt, non-CODEL astrocytomas among histologically confirmed LrGG (**Figure 4**, **Supplementary Table 2**). The T2-FLAIR mismatch sign with FLAIR of TI < 2,400 ms exhibited a positive predictive value (PPV) of 100% and a negative predictive value (NPV) of 75% for identifying *IDH*mt, non-CODEL astrocytomas among LrGG.

### Validation of the T2-FLAIR Mismatch Sign With FLAIR of TI < 2,400 ms in the TCIA/TCGA Dataset (Study From Cohort 3)

**Figure 5:**
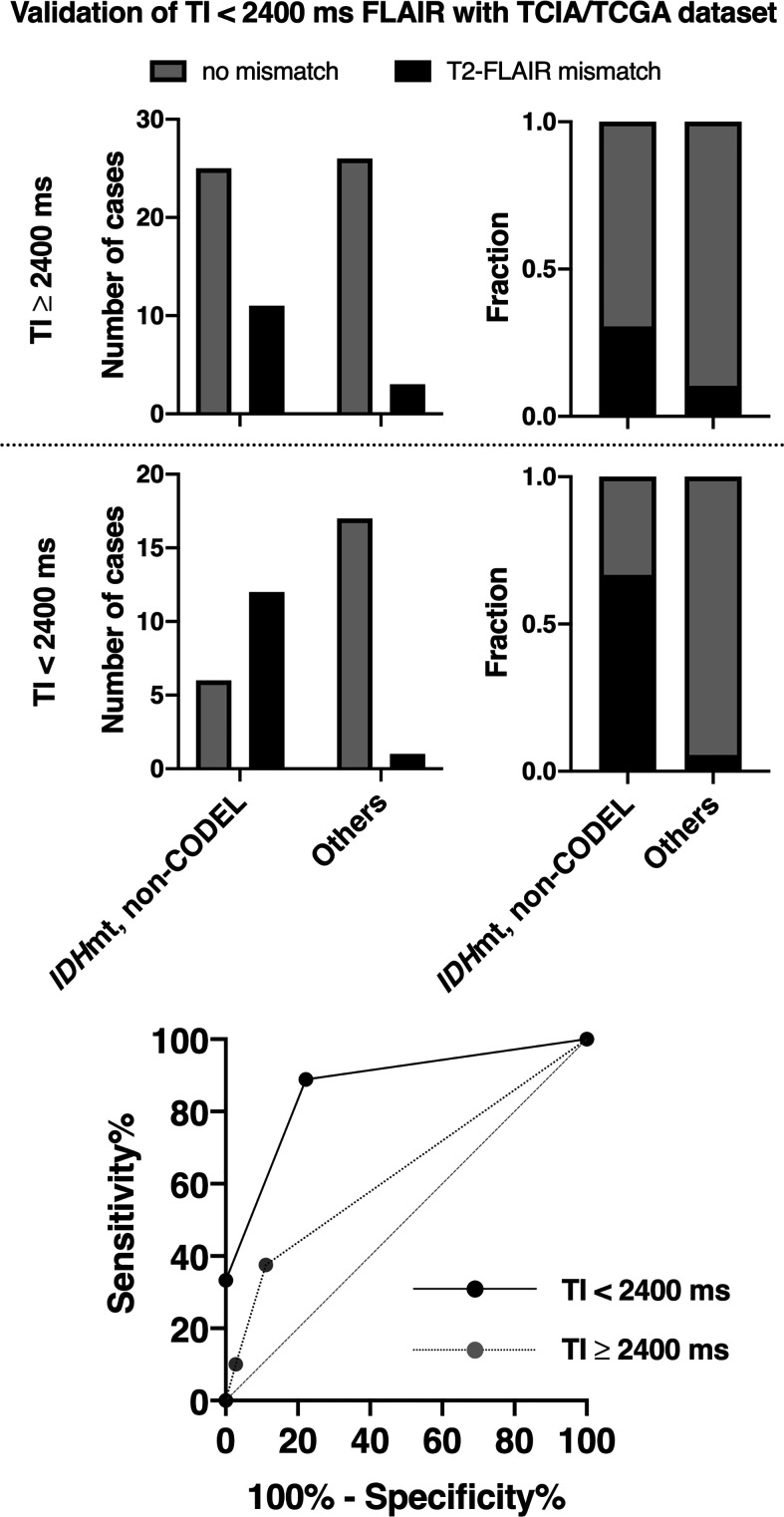
The figures show the frequencies of presence or absence of the T2-FLAIR mismatch sign found in the TCIA/TCGA LrGG cohort. We divided the cohort into two groups according to the TI for FLAIR acquisition. The T2-FLAIR mismatch sign was more frequently positive for or *IDH*mt, non-CODEL astrocytomas if we acquired FLAIR with TI shorter than 2,400 ms. The ROC curve for identifying *IDH*mt, non-CODEL astrocytomas is presented at the lower panel. The area under the curve improved from 0.63 to 0.87 87 if we acquired FLAIR with TI shorter than 2,400 ms.

Inter-rater reliability of two readers assessed by Cohen’s kappa coefficient was 0.32. For the scans conducted in the TCIA/TCGA dataset with FLAIR using TI ≥ 2,400 ms, the sensitivity, specificity, PPV, and NPV of the T2-FLAIR mismatch sign to identify *IDH*mt, non-CODEL astrocytomas were 30, 92, 80, and 54%, respectively. For the scans conducted with FLAIR using TI < 2,400 ms, those metrics improved to 67, 94, 92, and 74%, respectively. When we calculated the ROC curve for identifying *IDH*mt, non-CODEL astrocytomas, the area under the curve improved from 0.63 to 0.87 87 if we acquired FLAIR with TI shorter than 2,400 ms (**Figure 5**, **Supplementary Table 3**).

## Discussion

Although the benefit of presurgical knowledge of the molecular characteristics of LrGG is still debatable, pretreatment identification of genetic markers of LrGG could help both patients and clinicians to choose the most appropriate action to control the disease better. For example, suspicion of *IDH* being wildtype indicates poor prognosis (4, 5, 23), which encourages rapid and aggressive intervention, even at the expense of sacrificing the quality of daily life in some cases. On the contrary, if the *IDH* status is suspected to be mutant, one can expect a much favorable prognosis for the patient, and we could pursue a more careful balance between radical resection and preservation of the quality of daily life of the patient (24). The neuro-oncology and neuroradiology communities have made numerous attempts to develop non-invasive molecular diagnostic techniques for gliomas. These techniques include but are not limited to multiparametric MRI (7), diffusion-weighted image (DWI) (6), positron emission tomography (25), and radiomic analysis with the aid of machine learning (8, 9, 26–28). These reports claim to achieve approximately 80~85% diagnostic accuracy for identifying *IDH* mutation among LrGG. However, applying these methods into clinical practice is still challenging due to their relatively complex image analyzing procedures.

On the other hand, the T2-FLAIR mismatch sign is an image-based molecular diagnostic marker that clinicians can readily incorporate into clinical practice, as both T2WI and FLAIR are routine MRI protocols for gliomas. The T2-FLAIR mismatch sign is an imaging surrogate specific for *IDH*mt, non-CODEL astrocytomas, performance of which several studies have validated (13–15, 29–31). However, the sensitivity and NPV of the T2-FLAIR mismatch sign have been reported to be relatively low, as quite a few *IDH*mt, non-CODEL astrocytomas do not present the T2-FLAIR mismatch sign (13–15). Although a mechanistic explanation of the T2-FLAIR mismatch sign is challenging, our recent study revealed that *IDH*mt, non-CODEL astrocytomas often contain tumors that exhibit long T1 and T2 effects, which phenomenon could be the leading cause of the T2-FLAIR mismatch sign. Moreover, we demonstrated that tuning TI for FLAIR could increase the frequency of appearance of the T2-FLAIR mismatch sign in *IDH*mt, non-CODEL astrocytomas. Lowering the TI to 2,100 from 2,700 ms in 3T for the synthetic FLAIR reconstruction using T1- and T2-map significantly improved visualization of the T2-FLAIR mismatch sign (16). Taking advantage of this knowledge, we set a threshold of TI as 2,400 ms in 3T for acquiring FLAIR and studied the impact of different TIs on the diagnostic accuracy of the T2-FLAIR mismatch sign. As expected, FLAIR acquired with lower TI exhibited higher diagnostic accuracy both in our local dataset and in the TCIA/TCGA dataset used for validation purposes (**Figures 4** and **5**). NPV of the dataset with FLAIR acquired with TI longer than 2,400 ms was 51%, while that of FLAIR with TI shorter than 2,400 ms was 74%. More importantly, **Figure 3** illustrates the impact of TI on the presence or absence of the T2-FLAIR mismatch sign in the real clinical setting.

The value of the FLAIR sequence lies in the high lesion contrast by muting the background of the brain using an inversion recovery sequence (32). As the target tissue for signal suppression in FLAIR is often the CSF, FLAIR aims to suppress T1-relaxation time of 4,000 ms under 3T, which converts to TI of approximately 2,700 to 2,800 ms in 3T. For example, in stroke imaging, TI of 2,600 ms in 3T or 2,200 ms in 1.5T has been used to detect the DWI-FLAIR mismatch sign (33). Our research highlights the importance of tailoring the FLAIR sequence in glioma imaging by lowering TI rather than merely using the conventional FLAIR sequence developed for stroke and neuroinflammation imaging.

Some limitations should be mentioned in the end. Firstly, although shortening TI does increase the sensitivity of the T2-FLAIR mismatch sign, sensitivity is still approximately 70% at its best. Thus, the “absence of the T2-FLAIR mismatch sign” cannot exclude the possibility of the tumor not being *IDH*mt, non-CODEL astrocytomas. Secondly, due to the qualitative nature of the T2-FLAIR mismatch sign, inter-rater reliability was unstable, as Cohen’s kappa coefficient ranged from 0.32 to 0.60. In our series, rater 2 consistently showed a stricter interpretation of the T2-FLAIR mismatch sign. Careful MR reading by several experienced clinicians seems mandatory in order to correctly identify *IDH*mt, non-CODEL astrocytomas, even using short TI for FLAIR acquisition.

In conclusion, we provide evidence that TI for FLAIR acquisition significantly impacts the diagnostic accuracy of the T2-FLAIR mismatch sign, especially sensitivity and NPV. Although external cohorts should validate our findings, we propose that it is necessary for LrGG imaging to acquire FLAIR with TI shorter than 2,400 ms in 3T.

## Data Availability Statement

The original contributions presented in the study are included in the article/**Supplementary Material**. Further inquiries can be directed to the corresponding author.

## Ethics Statement

The studies involving human participants were reviewed and approved by the internal ethical review boards of Osaka University Graduate School of Medicine (approval number: 13244). The patients/participants provided their written informed consent to participate in this study.

## Author Contributions

MK, HA, MT, TU, JF, KIs, NKi, RH, NKa, KIc, YK, YN, and HK collected data. MS, AA, HT, and KN evaluated MR images. MK and HA analyzed MR data and genetic data. MK, HA, and HK wrote the manuscript. All authors contributed to the article and approved the submitted version.

## Funding

This research was funded by the Japan Society for the Promotion of Science (19K09526), Takeda Science Foundation, and MSD Life Science Foundation. All scientific grants were for MK.

## Conflict of Interest

The authors declare that the research was conducted in the absence of any commercial or financial relationships that could be construed as a potential conflict of interest.

## References

[1] International_Agency_for_Research_on_Cancer WHO Classification of Tumours of the Central Nervous System. Lyon, France: International Agency for Research on Cancer (2016).

[2] Cairncross G Wang M Shaw E Jenkins R Brachman D Buckner J Phase III Trial of Chemoradiotherapy for Anaplastic Oligodendroglioma: Long-Term Results of RTOG 9402. J Clin Oncol (2013) 31:337–43. 10.1200/jco.2012.43.2674 PMC3732012 23071247

[3] Buckner JC Shaw EG Pugh SL Chakravarti A Gilbert MR Barger GR Radiation plus Procarbazine, CCNU, and Vincristine in Low-Grade Glioma. New Engl J Med (2016) 374:1344–55. 10.1056/nejmoa1500925 PMC5170873 27050206

[4] Suzuki H Aoki K Chiba K Sato Y Shiozawa Y Shiraishi Y Mutational landscape and clonal architecture in grade II and III gliomas. Nat Genet (2015) 47:458–68. 10.1038/ng.3273 25848751

[5] Eckel-Passow JE Lachance DH Molinaro AM Walsh KM Decker PA Sicotte H Glioma Groups Based on 1p/19q, IDH, and TERT Promoter Mutations in Tumors. New Engl J Med (2015) 372:2499–508. 10.1056/nejmoa1407279 PMC4489704 26061753

[6] Leu K Perfusion and diffusion MRI signatures in histologic and genetic subtypes of WHO grade II–III diffuse gliomas. J Neuro-oncol (2017) 134(1):177–88. 10.1007/s11060-017-2506-9 PMC7927357 28547590

[7] Kickingereder P Sahm F Radbruch A Wick W Heiland S von Deimling A IDH mutation status is associated with a distinct hypoxia/angiogenesis transcriptome signature which is non-invasively predictable with rCBV imaging in human glioma. Sci Rep-uk (2015) 5:16238. 10.1038/srep16238 PMC4633672 26538165

[8] Fukuma R Yanagisawa T Kinoshita M Shinozaki T Arita H Kawaguchi A Prediction of IDH and TERT promoter mutations in low-grade glioma from magnetic resonance images using a convolutional neural network. Sci Rep-uk (2019) 9:20311. 10.1038/s41598-019-56767-3 PMC6937237 31889117

[9] Arita H Kinoshita M Kawaguchi A Takahashi M Narita Y Terakawa Y Lesion location implemented magnetic resonance imaging radiomics for predicting IDH and TERT promoter mutations in grade II/III gliomas. Sci Rep-uk (2018) 8:11773. 10.1038/s41598-018-30273-4 PMC6078954 30082856

[10] Andronesi OC Rapalino O Gerstner E Chi A Batchelor TT Cahill DP Detection of oncogenic IDH1 mutations using magnetic resonance spectroscopy of 2-hydroxyglutarate. J Clin Invest (2013) 123:3659–63. 10.1172/jci67229 PMC3754248 23999439

[11] Choi C Ganji SK DeBerardinis RJ Hatanpaa KJ Rakheja D Kovacs Z 2-hydroxyglutarate detection by magnetic resonance spectroscopy in IDH-mutated patients with gliomas. Nat Med (2012) 18:624. 10.1038/nm.2682 22281806 PMC3615719

[12] Andronesi OC Kim GS Gerstner E Batchelor T Tzika AA Fantin VR Detection of 2-Hydroxyglutarate in *IDH*-Mutated Glioma Patients by In Vivo Spectral-Editing and 2D Correlation Magnetic Resonance Spectroscopy. Sci Transl Med (2012) 4:116ra4–4. 10.1126/scitranslmed.3002693 PMC3720836 22238332

[13] Jain R Johnson DR Patel SH Castillo M Smits M van den Bent MJ ‘Real world’ use of a highly reliable imaging sign: ‘T2-FLAIR mismatch’ for identification of IDH mutant astrocytomas. Neuro-oncology (2020) 22(7):936–43. 10.1093/neuonc/noaa041 PMC7339896 32064507

[14] Broen MPG Smits M Wijnenga MMJ Dubbink HJ Anten MHME Schijns OEMG Bent MJ van den. The T2-FLAIR mismatch sign as an imaging marker for non-enhancing IDH-mutant, 1p/19q-intact lower-grade glioma: a validation study. Neuro-oncology (2018) 20:1393–9. 10.1093/neuonc/noy048 PMC6120363 29590424

[15] Patel SH Poisson LM Brat DJ Zhou Y Cooper L Snuderl M T2-FLAIR Mismatch, an Imaging Biomarker for IDH and 1p/19q Status in Lower-grade Gliomas: A TCGA/TCIA Project. Clin Cancer Res (2017) 23:6078–85. 10.1158/1078-0432.ccr-17-0560 28751449

[16] Kinoshita M Uchikoshi M Sakai M Kanemura Y Kishima H Nakanishi K T2-FLAIR Mismatch Sign Is Caused by Long T1 and T2 of IDH-mutant, 1p19q Non-codeleted Astrocytoma. Magn Reson Med Sci (2020). 10.2463/mrms.bc.2019-0196 PMC7952199 32101817

[17] Sasaki T Kinoshita M Fujita K Fukai J Hayashi N Uematsu Y Radiomics and MGMT promoter methylation for prognostication of newly diagnosed glioblastoma. Sci Rep-uk (2019) 9:14435. 10.1038/s41598-019-50849-y PMC6783410 31594994

[18] The_Cancer_Imaging_Archive (2020). Available at: https://public.cancerimagingarchive.net/ncia/login.jsf (Accessed February 1, 2020).

[19] The_Cancer_Genome_Atlas (2020). Available at: https://www.cancer.gov/about-nci/organization/ccg/research/structural-genomics/tcga (Accessed February 1, 2020).

[20] Arita H Yamasaki K Matsushita Y Nakamura T Shimokawa A Takami H A combination of TERT promoter mutation and MGMT methylation status predicts clinically relevant subgroups of newly diagnosed glioblastomas. Acta Neuropathol Commun (2016) 4:79. 10.1186/s40478-016-0351-2 27503138 PMC4977715

[21] Okita Y Nonaka M Shofuda T Kanematsu D Yoshioka E Kodama Y 11C-methinine uptake correlates with MGMT promoter methylation in nonenhancing gliomas. Clin Neurol Neurosur (2014) 125:212–6. 10.1016/j.clineuro.2014.08.004 25178915

[22] Ceccarelli M Barthel FP Malta TM Sabedot TS Salama SR Murray BA Molecular Profiling Reveals Biologically Discrete Subsets and Pathways of Progression in Diffuse Glioma. Cell (2016) 164:550–63. 10.1016/j.cell.2015.12.028 PMC4754110 26824661

[23] Arita H Narita Y Fukushima S Tateishi K Matsushita Y Yoshida A Upregulating mutations in the TERT promoter commonly occur in adult malignant gliomas and are strongly associated with total 1p19q loss. Acta Neuropathol (2013) 126:267–76. 10.1007/s00401-013-1141-6 23764841

[24] Kawaguchi T Sonoda Y Shibahara I Saito R Kanamori M Kumabe T Impact of gross total resection in patients with WHO grade III glioma harboring the IDH 1/2 mutation without the 1p/19q co-deletion. J Neuro-oncol (2016) 129:505–14. 10.1007/s11060-016-2201-2 27401154

[25] Li L Mu W Wang Y Liu Z Liu Z Wang Y A Non-invasive Radiomic Method Using 18F-FDG PET Predicts Isocitrate Dehydrogenase Genotype and Prognosis in Patients With Glioma. Front Oncol (2019) 9:1183. 10.3389/fonc.2019.01183 31803608 PMC6869373

[26] Beig N Bera K Prasanna P Antunes J Correa R Singh S Radiogenomic-Based Survival Risk Stratification of Tumor Habitat on Gd-T1w MRI Is Associated with Biological Processes in Glioblastoma. Clin Cancer Res (2020) 26:1866–76. 10.1158/1078-0432.ccr-19-2556 PMC7165059 32079590

[27] Lu C-F HSU F-T Hsieh KL-C Kao Y-CJ Cheng S-J Hsu JB-K Tsai P-H Machine Learning-Based Radiomics for Molecular Subtyping of Gliomas. Clin Cancer Res (2018) 24(18):4429–36. 10.1158/1078-0432.ccr-17-3445 29789422

[28] Lohmann P Lerche C Bauer EK Steger J Stoffels G Blau T Predicting IDH genotype in gliomas using FET PET radiomics. Sci Rep-uk (2018) 8:13328. 10.1038/s41598-018-31806-7 PMC6127131 30190592

[29] Juratli TA Radiographic assessment of contrast enhancement and T2/FLAIR mismatch sign in lower grade gliomas: correlation with molecular groups. J Neuro-oncol (2019) 141:327–35. 10.1007/s11060-018-03034-6 PMC6924170 30536195

[30] Batchala PP Muttikkal TJE Donahue JH Patrie JT Schiff D Fadul CE Neuroimaging-Based Classification Algorithm for Predicting 1p/19q-Codeletion Status in IDH-Mutant Lower Grade Gliomas. Am J Neuroradiol (2019) 40:426–32. 10.3174/ajnr.a5957 PMC7028667 30705071

[31] Goyal A Yolcu YU Goyal A Kerezoudis P Brown DA Graffeo CS The T2-FLAIR–mismatch sign as an imaging biomarker for IDH and 1p/19q status in diffuse low-grade gliomas: a systematic review with a Bayesian approach to evaluation of diagnostic test performance. Neurosurg Focus (2019) 47:E13. 10.3171/2019.9.focus19660 31786548

[32] Hajnal JV Bryant DJ Kasuboski L Pattany PM Coene BD Lewis PD Use of Fluid Attenuated Inversion Recovery (FLAIR) Pulse Sequences in MRI of the Brain. J Comput Assist Tomo (1992) 16:841–4. 10.1097/00004728-199211000-00001 1430427

[33] Thomalla G Cheng B Ebinger M Hao Q Tourdias T Wu O DWI-FLAIR mismatch for the identification of patients with acute ischaemic stroke within 4·5 h of symptom onset (PRE-FLAIR): a multicentre observational study. Lancet Neurol (2011) 10:978–86. 10.1016/s1474-4422(11)70192-2 21978972

